# Family members’ perceptions of end-of-life care across diverse locations of care

**DOI:** 10.1186/1472-684X-12-25

**Published:** 2013-07-20

**Authors:** Romayne Gallagher, Marian Krawczyk

**Affiliations:** 1 Division of Palliative Care, Department of Family and Community Medicine, Providence Health Care, University of British Columbia; 2 Department of Sociology & Anthropology, Simon Fraser University, Burnaby, BC, Canada

**Keywords:** Family satisfaction, End-of-life care, Palliative care, Acute care, Hospice, Residential care, Bereaved relative

## Abstract

**Background:**

The goal of the study was to assess perceived level of satisfaction with end-of-life care, focusing on the last 48 hours of life.

**Methods:**

A previously validated instrument was used in a telephone survey with bereaved family members (n=90) of patients who died within an organization in British Columbia.

**Results:**

Bereaved family members had many unmet needs for information about the patient’s changing condition, the process of dying, how symptoms would be managed and what to do at the time of death. In addition, many bereaved relatives felt that the patient or resident had an unmet need for emotional support and that their own emotional needs were not addressed adequately. The last place of care had the most significant effect on all of these variables, with acute care and residential care having the most unmet needs. Hospice had the fewest unmet needs, followed by the palliative and the intensive care units.

**Conclusions:**

We discuss these findings in relation to overall satisfaction with care, focus on individual, ethno-cultural and diversity issues, information and decision-making, symptom management and attending to the family. We conclude by offering possible practices address the end-of-life needs of patients and family members.

## Background

Improving the quality of health care for patients at the end of their lives has become a major national, clinical, and research objective [1,2]. Increasingly, end-of-life (EOL) care is receiving the attention of policy makers and the public, influencing debates on physician-assisted suicide shaping their expectations for care at the end of life [3].

Models of EOL care [4] have been developed from multiple qualitative and quantitative studies. In turn, questionnaires to assess the dying experience in bereaved relatives have been validated [5] and used to measure care [6]. A recent review of the literature of family satisfaction with EOL care [7] has identified key domains of EOL care: Patient comfort and symptom management, emotional support, information and education; communication and competence have been identified by quantitative studies. Qualitative studies have revealed more complex indicators such as care provider response and time with patient; providers’ interactional approach; service coordination, consistency and flexibility; personalized and individual care; and the facility environment. This research has been key in informing what is quality health care for people near the end of life.

Family most often become caregivers for the patient prior to and during an admission to a healthcare facility. Suboptimal physical and mental health has been reported in caregivers [8,9]. Prolonged grieving, or complicated grief, has been associated with lack of preparedness for the death [10]. Earlier access to palliative care services in the US has been associated with a positive affect on the post-death health of the surviving spouse [11].

As part of a strategy to improve access and build capacity for palliative care across the organization Providence Health Care (PHC) Palliative Care Program undertook a bereaved relative interview study. The clinical perception was that the quality of care in the last two days of life was variable across the organization and this study was a way to inform this perception and provide data to inform improvement.

PHC is a faith-based health care organization that includes two acute care facilities (500 and 100 beds), a hospice (12 beds) and five residential care sites (686 beds in total). The palliative care program is an outreach and consult model that is accessible from anywhere in the organization. The outreach team consists of an advance practice nurse and a physician. Any healthcare provider, patient or family member may call the team for assistance with care.

The palliative team provides primary care in the hospice and on a 12-bed palliative care unit in the larger acute care hospital. The majority of the deaths occur in residential care and acute care in individuals, who may or may not, have accessed the palliative care team. If an organization is to embrace palliative care as a core service provided to all patients with life-limiting illnesses then adequate EOL care should be available across the organization. In addition, PHC is a teaching site for medical, nursing and other health science schools.

## Methods

Based on the estimate of 1100 deaths per year across the organization, a statistician determined the approximately 100 deaths should give a fair snapshot of the last two days of life in a PHC facility. Next of kin records were drawn from all patient deaths within PHC between September 3rd 2009 and March 30th, 2010. Hospice records were taken from April 4th through October 10th 2010. Patients who were under the age of 18, died from trauma, or who died before being admitted into the hospital were excluded. From the 553 potential participants, 225 entries were invalidated due to: 1) in care less than 48 hours; 2) next of kin and emergency contact outside of province, or 3) no contact or incomplete information.

An invitational letter was mailed to the remaining 332 potential participants and if they were thought to speak either a Chinese language or Punjabi they were sent a letter in that language. That letter informed non-English speakers that the interview would be conducted with a professional interpreter as well as the interviewer. Potential participants were notified of a 1–800 number they could call if they did not want any further contact. Ten business days later, those who did not call the refusal phone line were contacted by the interviewer for a total of three attempts over a one-month period. The interviewer assessed inclusion and exclusion criteria and arranged to conduct the interview. 146 people did not respond to the phone message. Informed consent was obtained for each participant.

Participants had to speak English, Cantonese, Mandarin or Punjabi and attended to the patient during the last weeks of the patient’s life as well as had experience with the patient’s health care providers in the last forty-eight hours of life. Bereaved relatives who did not meet the above criteria, were experiencing acute emotional distress, or were suspected of having cognitive issues such as dementia were excluded. Employees of PHC were also excluded. Ten potential participants were excluded by these criteria.

Of the 292 remaining potential participants, thirty could not be contacted from the information provided or had moved with no forwarding information, 35 refused to participate once contacted, 6 felt they were not well enough to participate, 4 stated the patient received good or excellent care but felt that discussing it further would be too painful, and 3 sent back consents but the interviewer was unable to contact them. A total of 90 interviews were conducted.

The After-Death Bereaved Family Member Interview (ADBFMI) is a multi-domain survey tool that examines seven domains of end-of-life care within hospitals, hospices, and residential care facilities, including:

•Physical and emotional support

•Inform and promote shared decision-making

•Encourage advance care planning

•Focus on individual

•Attend to the emotional and spiritual needs of the family

•Provide coordination of care

•Support for the self-efficacy of the family

The questionnaire is able to measure process as well as outcomes of care, and has been validated and used in quality improvement studies in Canada and United States [4,12-14]. The survey contains 38 questions that focus on measuring whether the care provided in the above domains met the needs and expectations of family members, along with an overall measure of their satisfaction within these domains. The reliability varies from 0.58 to 0.87 and the validity ranges from 0.36–0.69 which was thought to be adequate for a tool intended for quality improvement of the care of the dying [4]. This survey tool was selected because it had questions specific to the last place of care, was multidimensional and included acute care, hospice and residential care.

Our study added seven open-ended questions, one to the end of each overall ranking question, and asked participants to elucidate their reasons for the ranking. The purpose was to understand, in the participants’ own words, the connection between the numerical ranking and the experience of care. The narrative data was captured in hand-written notes. Provisional analysis occurred through grouping responses based on the question prompt.

An Excel worksheet from the survey author automatically calculated problem scores, scale scores, and domain scores. Chi-square tests were done for specific variables of: last location of care, patient age, respondent age, patient gender, respondent gender, ethno-cultural heritage of respondent, and length of stay in correlation to perceived levels of satisfaction and specific domain questions. ANOVA tests were also used to compare the means within these variables. Narrative responses were transcribed verbatim with sample passages extracted to represent the rich descriptions that emerged.

The study was approved by Providence Health Care’s Research Ethics Board.

## Results

After reporting the participant demographics and the overall ranking of care and the domain scores, we report further on the associations of factors with the scores and on specific issues that warranted improvement.

Patient characteristics are in Table 1. Ninety interviews occurred making the response rate, 31.5%, similar to other after-death survey rates [15]. Respondents were close family members in almost all instances with 44% being a child, 40% a spouse, 2.5% a sibling, 2.5% a parent, 5% a more distant relative and 6% a friend. Almost two thirds of respondents were female and the average age of both male and female respondents was 60 years. Twenty percent of the respondents self-reported their own health as fair or poor. Interviews lasted approximately 30 minutes (range 17 to 75 minutes).

**Table 1:** Demographics of deceased patients

**Last place of care:**	**Percentage (number of participants)**
Palliative care unit	27% (24)
Hospice	10% (9)
Acute Care large	19% (17)
ICU	10% (9)
Acute Care small	20% (18)
Residential	14% (13)
**Gender:**	
Male	43% (39)
Female	57% (61)
**Age:**	
Average age	77.5 years
Median age	81 years
**Length of stay:**	
Total average:	84 days
Hospital patients	22 days
Hospice patients	17 days
Residential care	1.2 years
**Average age of patients in relation to location of care:**	
Palliative Care Unit	70 years
ICU	72 years
Acute care large	77 years
Acute care small	86 years
Residential	86 years
Hospice	75 years
**Most responsible cause of death:**	
Cancer	26% (24)
Organ failure	14% (13)
Cardiac	12% (11)
Dementia	8% (7)
Stroke	8% (7)
Other	32% (28)
**Ethno**-**cultural heritage:**	
Canadian	58% (52)
European	19% (18)
East Asian	8% (7)
South Asian	6% (5)
Other	9% (8)
**Marital status:**	
Married	46% (41)
Widowed	36% (33)
Single	11% (10)
Divorced	7% (6)
**Educational level:**	
< High school	29% (26)
High school	22% (20)
Technical school	12% (11)
College graduates	17% (15)
Advanced degree	20% (18)
**Living situation:**	
Living alone	27% (24)
Living w/others	73% (66)
**Religious affiliation:**	
None	39% (35)
Catholic	26% (24)
Protestant/ Anglican/ United or Presbyterian	16% (14)
Sikh	7% (6)
Non-denominational Christian	4% (4)
Other	8% (7)
**Location of care preceding final admission:**	
Home	42% (38)
Hospital	31% (28)
Residential care	15% (14)
Other	11% (10)

Comparing those who chose not to participate with respondents there were significantly more male patients whose care giver chose not to respond (p=.02). Non-respondents were more than four times likely to be a caregiver of a patient in a facility for more than 180 days. Caregivers of patients in a palliative care setting were more likely to respond than those in residential care or the ICU (p=.03).

The study did not directly ask if the Palliative Outreach and Consult Team was involved in the patient’s care because we were following the ABDFMI instrument, but many respondents mentioned being exposed to the team and benefiting from their support.

“The nice lady from palliative care was wonderful; she wanted us to come to palliative care and I said ‘[My mother] is not there yet’ and the lady respected that. She came back a couple more times; she was the only one who told me what it was going to be like in the final stages. Otherwise they didn’t outline for me what was going to take place—and I’d never been through that. I would have wanted more of that.”

The overall ranking of care and the domain scores are presented in Table 2.

**Table 2:** Overall ranking and domain scores

**Overall ranking scale (on a scale from 1–10)**	
**Question: In the last two days of care, how well did the doctors, nurses and other professional staff…**	**Average**
Provide medical care that respected his/her wishes?	8.2
Make sure that [patient] died with dignity – that is died on her/his own terms?	8.2
Make sure [patient’s] symptoms were controlled to a degree acceptable to her/him?	8
Communicate with patient and family about the illness and likely outcomes of care?	7.3
Provide emotional support for you and [patient’s] family and friends?	6.8
What number would you give the overall care that [patient] received in the last two days of life?	7.7
**Scale score**	**7.7**
**Comparison of Domain Scores (range: 0 to 1)**	
Advance care planning	0.10
Coordination	0.16
Focus on individual	0.27^1^
Informing & decision making	0.30^1^
Attend to family	0.51^1^

^1^ An overall mean problem score or domain score greater than 0.20 is indicative of an important opportunity to improve the quality of care.

Length of stay was statistically significant when comparing admission lengths 4–21 days (74%) and 22–60 days (60%) for respondent’s rate of “very satisfied” with overall care, though the data set is small. Neither patient nor respondent gender seemed to effect overall satisfaction with care. Respondents of older patients expressed lower satisfaction, although due to small sample size this did not reach significance. Neither patient nor respondent gender impacted overall satisfaction rating. Ethno-cultural differences existed, with South Asians expressing lowest satisfaction ratings (50%) versus a high of 77% among Europeans. Again, while these findings had statistical strength, sample size does not allow for further analysis.

Last place of care significantly affected response to the overall satisfaction with care in the last two days of life. Table 3 shows the questions that elicited some of the most significant differences between last place of care.

**Table 3:** Selection of questions from ADBFMI and last place of care

**Last place of care**							
	PCU	ICU	Acute care small	Residential care	Acute care large	Hospice	T0TAL
	24	9	18	13	16	9	89
**What number would you give the overall care that** [**patient**] **received in the last two days of life?**							
Not satisfied (0-5/10)	8% (2)	11% (1)	17% (3)	31% (4)	31% (5)	0	17% (15)
Satisfied/somewhat satisfied (5-7/10)	8% (2)	11% (1)	22% (4)	31% (4)	13% (2)	11% (1)	16% (14)
Very satisfied (8-10/10)	84% (20)	78% (7)	61% (11)	38% (5)	56% (9)	89% (8)	67% (60)
**In the last two days of care, how well did the doctors, nurses and other professional staff provide medical care that respected his/her wishes?**							
Not satisfied	4% (1)	11% (1)	0	23% (3)	25% (4)	0	10% (9)
Satisfied/somewhat satisfied	13% (3)	0	17% (3)	23% (3)	25% (4)	0	15% (13)
Very satisfied	83% (20)	89% (8)	83% (15)	54% (7)	50% (8)	100% (9)	75% (67)
**In the last two days of care, how well did the doctors, nurses and other professional staff make sure that [patient] died with dignity – that is died on her/his own terms?**							
Not satisfied	8% (2)	0	22% (4)	23% (3)	25% (4)	0	15% (13)
Satisfied/somewhat satisfied	8% (2)	11% (1)	0	8% (1)	25% (4)	0	9% (8)
Very satisfied	84% (20)	89% (8)	78% (14)	69% (9)	50% (8)	100% (9)	76% (68)
**In the last two days of care, how well did the doctors, nurses and other professional staff make sure [patient’s] symptoms were controlled to a degree acceptable to her/him?**							
Not satisfied	13% (3)	11% (1)	6% (1)	23% (3)	18% (3)	0	12% (11)
Satisfied/somewhat satisfied	25% (6)	0	11% (2)	31% (4)	13% (2)	22 % (2)	18% (16)
Very satisfied	63% (15)	89% (8)	83% (15)	46% (6)	69% (11)	78% (7)	70% (62)
**Would you have wanted (some/more) information about what to expect while (he/she) was dying?**							
No	63% (15)	67% (6)	44% (8)	54% (7)	50% (8)	56% (5)	55% (49)
Yes	38% (9)	33% (3)	56% (10)	46% (6)	50% (8)	44% (4)	45% (40)
**In (patient’s) last two days of care, how often were you or other family members kept informed about (patient’s condition) – always , usually, sometimes, never? **							
Always	38% (9)	78% (7)	39% (7)	39% (5)	38% (6)	56% (5)	44% (39)
Usually, sometimes, or never	63% (15)	22% (2)	61% (11)	62% (8)	63% (10)	44% (4)	56% (50)
**In the last two days of care, how well did the doctors, nurses and other professional staff provide emotional support for you and [patient’s] family and friends?**							
Not satisfied	17% (4)	22% (2)	33% (6)	39% (5)	38% (6)	0	26% (23)
Satisfied/somewhat satisfied	29% (7)	22% (2)	11% (2)	23% (3)	19% (3)	22% (2)	21% (19)
Very satisfied	54% (13)	56% (5)	56% (10)	39% (5)	44% (7)	78% (7)	53% (47)

Overall, the lowest rankings were for “communicating with the family” and “providing support to the patient’s family and friends”. Looking at the domain scores the area that needed the most improvement was “attending to the family” followed by “focus on the individual” and “information and decision-making”.

### Focus on individual needs

Respondents expressing concern for patients’ personal care needs reached a high of 77% in residential care, compared to the palliative care unit (PCU) and (MSJ) at 33% and ICU at 22% (p<0.0001). In the larger acute care (SPH) 63% of respondents expressed concern regarding personal care significantly different when compared to the palliative unit in the same hospital. (p=0.003). The lowest rate of concern was at hospice with only 11% concern and was significant when compared with the palliative care unit (p=0.0005). Respondents less than 50 years reported a substantially higher level of concern than respondents 66–79 years (p=0.0007). Reports of concern increased with length of stay but this finding did not reach statistical significance.

Ethno-cultural and diversity issues were also raised, particularly around food, co-ed rooms, recognition of same-sex partners, and gender of nurses. Particularly for patients of South Asian ethno-cultural heritage the food provided was often seen as culturally inappropriate and there were concerns about having female patients in mixed gender rooms and cared for by male nurses.

“It was a four person room with both men and women. It should be segregated, and there’s only a small thin curtain. Because she is a lady she should be looked after by another lady; this is especially important because of the cultural difference due to her age, specifically around bathing and dressing. She wasn’t comfortable with the male nurses.”

Thirty-one of ninety respondents stated that patients had feelings of sadness and/or anxiety in the last 48 hours of life. Older respondents were much more likely to report no patient anxiety or sadness, or not knowing if the patient was anxious or sad in the last 48 hours of life though this was not statistically significant. Respondents of female patients more often reported “no” or “don’t know” compared to respondents of male patients (72% versus 59%) (p=0.03) For patients that were perceived as experiencing anxiety or sadness, respondents stated overwhelmingly (90%) that there was no, or not enough, help to support patients in this aspect.

“I wish I had known sooner [that she was dying] because maybe we could have helped her during this process when she was so upset. I didn’t have a chance to talk about any of these feelings and explore with her. It was lacking and I felt very vulnerable. I wish that there was somebody we could have talked to about all of that; and have it more of a spiritual experience.”

One man expressed deep concern that although he was legally married to his male spouse, and had legal decision-making power, advance care planning discussions were focused solely on the patient’s sister. Another gay male expressed profound gratitude to the palliative unit for the staff’s recognition of his personal relationship to the patient even though it had not been formalized through marriage.

### Informing and decision-making

The majority of respondents asserted that the doctors listened to their concerns about the patient’s medical treatment (87%), and that relatively few care decisions were made about without adequate input from the family (11%).

“Everybody was so accessible—the floor was amazing. They were always there with a pat on the shoulder and a hug—even the doctors. The doctors were A+; he wasn’t a patient to them, he was a person.”

Yet communication emerged as the largest concern. 57% of respondents felt that they were not always informed about the patient’s condition, and 30% felt they received less information than was needed from physicians about the patient’s medical condition. No respondent stated that they received too much information about the patient’s condition.

“You know in the back of your mind that things can happen so you prepare yourself but you don’t expect it. So I’m not sure I would have wanted to know. But on the other hand, nobody really talked to me about it.”

“He wasn’t in the same state as they told me on the phone—they said he was ‘comfortable’ but he didn’t seem to be. Resting comfortably really meant being comatose. They should have mentioned that he was in a different state.”

Almost half of respondents (46%) reported desiring more information about the dying process. Respondent ethno-cultural heritage had strong significant in this response, with South Asian (83%) and European respondents (62%) desiring more information than Canadians (38%), but numbers of minority respondents were small. Even in hospice almost half of respondents stated they would have liked more information.

Respondents who spoke with the patient’s doctor in the last two days of care, felt they had difficulty in understanding what was to be expected from treatment (22%), or wanted further information about medications (23%).

“If you’re doing a study about this, tell them that family members need to know about the medicine and about death. Nobody wants to talk about it, but it’s there. Why does nobody talk when it’s clear that the person only has a couple of days?…Maybe if you are too open, people will panic. And maybe they don’t know themselves, so that’s a difficult balance. However, one nurse said ‘Why don’t you ask her if she’s afraid of dying?’ so I did and she nodded her head so the nurse called a priest and she settled right down. So that nurse was really helpful. When we asked we always got good information but usually we didn’t know how or what to ask.”

Despite the perceived lack of communication some respondents recognized that they were not always a willing partner in this exchange of information:

“We didn’t want to hear that he was going to die or when, so the staff had to dance around that information while providing care.”

“One nurse said ‘you know he is going to die’, which completely snapped me out of my oblivion. I didn’t appreciate it at the time but in retrospect I really did—it allowed me to prepare for the eventualities. I talked with the doctors and they thought he’d be fine just a few days before that.”

### Symptom management

The majority of respondents felt that communication about, and provision of, physical comfort delivered by Providence staff was excellent, good or adequate. While very few respondents reported concern regarding conflicting clinical communication about physical symptom management for pain (4%), more than a quarter (29%) of respondents stated that information about pain treatment was presented in a way they had difficulty understanding [16].

Twenty-one percent felt patients either received too much or too little amount of medication for pain. Many of these respondents indicated that poor pain control was due to patient under-reporting, or miscommunications between staff, rather than an issue of neglect or disbelief. However, several respondents also recounted troubling moments where patients expressed ongoing and severe pain that was not addressed. Others spoke about their reticence to question the care, for fear of negatively impacting the patient’s future care.

“Most nurses were really good about PRN meds—but one wasn’t. She said ‘Your mother needs to ask God to forgive her for all of her sins.’ I said ‘Fine, you want to watch her die in pain?’”

Almost one in four respondents (24%) felt that not enough help with breathing was given.

### Attending to the family

Thirty percent of respondents felt they did not receive the support they needed in dealing with difficult feelings during the last two days of the patient’s life. Of those who felt supported, nurses emerged as important sources for respondents’ emotional care, communication and support. Social workers were also seen as a valuable source of support for the majority of those who had interactions with them. When negative comments were about these professionals, most were made due to lack of knowledge about social work roles and/or system issues such as heavy workload.

A significant minority of participants expressed frustration with the lack of a follow-up phone call by a social worker or nurse, or an expression of condolence from the primary physician, unless there were significant unresolved medical questions regarding cause of death. This was even seen in patients in hospice or the palliative care unit.

“I thought maybe the doctor could have phoned me the day my mother died. I was in shock—I didn’t know what was cause of death, or if she went peacefully. I didn’t know if it was up to me to ask those questions? The staff were nice when I got there but they didn’t share any information with me.”

During the last two days of life only 28% of respondents reported having adequate contact with regards to their religious or spiritual beliefs. Residential care and ICU reported the highest levels of contact and the lowest was the non-palliative acute care.

Another aspect of attending to the family was care after the patient had died. Over one third of the patients wanted more information about what to do at the time of death (37%).

“I was left alone; when he died I walked to the nurses’ station but no one was there, so I walked back to the room, packed up a few things and left. I was all alone; someone should have stuck with me. I was out of my mind for a few moments, and I went home alone.”

## Discussion

Bereaved family members had many unmet needs for information about the patient’s changing condition, the process of dying, how symptoms would be managed and what to do at the time of death. In addition, bereaved relatives felt that the patient/resident had an unmet needs for personal care and emotional support and that their own emotional needs were not addressed adequately. Last place of care had an effect on all of these variables with acute care and residential care having the most unmet needs. Hospice was the place where the fewest unmet needs were followed by the palliative care unit and the intensive care unit.

Research using the ADBFMI instrument in other healthcare organizations has found similar EOL care concerns [6,9]. Other research using this instrument has highlighted how acuity of care is not related to perceptions of EOL care; rather that palliative care is strongly associated with higher rates of satisfaction with EOL care [6,17]. Again, similar to these findings, other studies using the ADBFMI instrument have found lowest rates of satisfaction with end-of-life care within residential care settings [16].

Palliative care is an approach to care with defined skills and knowledge that increases patient and family member satisfaction [17], reduces pain [18] and other symptoms but also reduces hospital admissions [19], costs and length of stay [20] and may increase length of life [21,22] Research shows that emphasis on the “unit of care” as being both patient and their identified family can positively impact patient and family member satisfaction with the emotional and spiritual support provided by their health care team at the very end of life, even if they are not receiving care on a specialized palliative unit [15,23,24].

There continues to be a gap between health care that honors palliative care principles and the health care we provide, even in palliative care settings. This study highlights the complexity of personal, environmental, and relational factors that hinder doctors, nurses and allied health staffs’ ability to provide quality end of life care, as well as issues that arise for next-of-kin. There are many reasons for these including system issues, lack of focusing on the goals of the patient and family, and provider’s lack of insight into what a family can be expected to know about the dying process.

One could be tempted to draw a direct relationship between satisfaction with end of life care and staffing as ICU and palliative care units tend to have higher staffing ratios than general medical wards and certainly higher than residential care. However, the hospice in many domains, including support to the family, managed to have higher satisfaction scores despite lower staffing ratios. Hospice may be more successful because staff have more time available for supportive listening due to few investigations and less drug administration. In hospice the goals of the patient and family are aligned with the care provided prior to the patient going to hospice. Patients and families may be self selecting to a form of care that meets their needs and therefore they are more satisfied. Symptom management and support in hospice is rated higher even than the palliative care unit likely because the unit is reserved for patients with the most difficult symptoms, physical and psychosocial.

The comments made by families also highlight one of the key complexities of family support—how families choose to interpret what care their loved one is or is not given [7]. Many families will interpret positive or negative experiences of caring “for” as the more personal caring “about” the person—something that many caregivers do not keep uppermost in mind when they are dealing with patients and families.

Providence Health Care is working on specific strategies to address the findings of this study. In residential care, a palliative care nurse is undertaking a pilot study of screening all admission to one facility to determine if an urgent or routine referral to palliative care is appropriate. This same nurse is exploring the concept of an “embedded” palliative care nurse within the facility to help guide staff in developing resident and family goals of care and care plans appropriate for end-of-life. A retrospective chart review of residents who died within six months of admission to residential care is in process to determine if goals of care were discussed and documented in order to provide evidence for quality improvement.

To improve EOL care in acute care we have developed a multi-professional group of champions of palliative care. This group will guide development of specific strategies to address gaps in care. Specific information about the dying process exists but staff must make the effort to point this out and be available for questions and support. We have built this information into terminal care orders with direction to the physician or nurse to ensure that the family has them. An automatic referral to social work and spiritual care now occurs with the terminal order set to improve support for the patient and family. Recent guidelines for psychosocial support [25] have been published and these could be used to build family assessment and support into the assessment and care of the patient.

In the large hospital (SPH) medicine ward a pilot study of screening each admission to identify who might benefit from a palliative approach to care has been started. It is hoped that identifying patients earlier and facilitating goals of care discussions may lead to the patient and family being better informed and supported.

A Palliative Care Awareness Week for the whole organization has occurred. Different educational and awareness events are planned for each site and adapted to the particular needs and culture of the site. Tools to assist providers in identifying patients who could benefit from a palliative approach to care and to assist in symptom assessment and management are available. We also have a website with detailed information about accessing palliative services, guidelines for symptom management and educational videos. Our team has incorporated this information and communication suggestions into an app that works on any smart phone.

The need for families to be supported after the death of a patient or resident was clear in this study. Educating health care providers to inform and support families until they leave the unit after the death is essential in order to help a family to feel cared for and not abandoned. A project is underway to develop interactive educational videos for staff but also video information for families about the natural process of dying.

Bereavement follow up has long been seen as part of palliative care yet it is never funded as a core service of chronic disease management. Identifying those at risk for complex bereavement could be combined with the assessment of family support needs with those families being recommended to specific resources in the community or as part of the organizational palliative care program. For those less complex, bereavement follow up could be adopted by adequately trained volunteers with support from spiritual care and social work.

### Limitations of the study

With 1100 deaths per year, there is a relatively high margin of error (9%) with a 95% confidence level. While many of these numbers reached strong statistical significance in our study, the sample size is too small to make causal arguments. Many participants stated that their perceptions were affected by events before the 48-hour time period and admitted that this shaped their responses. Respondents desired to provide differential scores between physicians, nurses, and allied support staff which the survey was not designed to do. A limitation of all studies using proxy estimation of what the patient experiences is that proxies draw on a different knowledge base and may have different understandings of the symptom than patients or providers. This is particularly relevant when evaluating less objective symptoms such as anxiety, depression and to a lesser extent pain [26]. The significant difference between potential participants and respondents would be interesting to follow up as this may more fully inform the quality of EOL care in the organization.

The ADBFMI lacks ethno-culturally specific questions that may pick up the potentially serious differences in perception of care. While the instrument looks at satisfaction with care in the last 48 hours many participants stated that their perceptions were affected by events before this time period, and admitted that this shaped their responses. Respondents desired to provide differential scores between physicians, nurses, and allied support staff which the survey was not designed to do. Several questions were not sensitive enough in design; for example, the combined response of “no” and “don’t know” to the query “Did the patient have any feelings of anxiety or sadness?” did not allow us to record differential responses to this question.

A limitation that has persisted despite the availability of interpreters is the lack of participation by those who do not speak English. This is particularly relevant since reporting of anxiety, not meeting personal care needs and not having enough information was significantly different between those of Canadian and non-Canadian ethnicity. Perhaps accessing bereaved relatives through cultural brokers may be a strategy that will enable more to participate in the study.

## Conclusions

There are significant unmet needs in dying patients and their families. This study highlights the areas in which organizations can improve the quality of care for those who are dying and for their families. It is essential that health care organizations and providers take steps to close these gaps so that all Canadians receive quality palliative care as part of their life-threatening illness.

## Competing interests

The authors declare that they have no competing interests.

## Authors’ contributions

RG conceived the project, participated in ethics submission and coordination of the study and drafted the manuscript. MK participated in ethics submission, carried out patient recruitment and the interviews and the data analysis and drafted the manuscript. Both authors have read and approved the final manuscript.

## Pre-publication history

The pre-publication history for this paper can be accessed here:

http://www.biomedcentral.com/1472-684X/12/25/prepub
